# Tobacco smoking is associated with cutaneous squamous cell carcinoma but not with basal cell carcinoma or melanoma in adult subjects at risk of skin cancer: A cross-sectional study

**DOI:** 10.18332/tid/185299

**Published:** 2024-05-14

**Authors:** Ilmari Uotila, Hanna Siiskonen, Salla Haimakainen, Ilkka Harvima

**Affiliations:** 1 Department of Dermatology, Kuopio University Hospital, University of Eastern Finland, Kuopio, Finland

**Keywords:** melanoma, tobacco smoking, squamous cell carcinoma, skin cancer, basal cell carcinoma

## Abstract

**INTRODUCTION:**

The relationship between tobacco smoking and cutaneous photodamage or malignancies is still unclear. In addition to smoking, both ultraviolet radiation and immunosuppression have an impact on carcinogenesis. The purpose was to study the association of smoking with cutaneous photoaging, actinic keratosis (AK), skin cancers, and pigment cell nevi in adult subjects at risk of any type of skin cancer.

**METHODS:**

In this cross-sectional study at Kuopio University Hospital, Finland, between May 2017 and October 2020, 488 subjects (aged 21–79 years, 246 males and 242 females, 94 with immunosuppression) were examined for a variety of skin lesions, photoaging severity, nevi, tobacco pack-years (TPY), as well as for possible confounding factors.

**RESULTS:**

In logistic regression analyses, no marked association was found between TPY and total skin photoaging, facial photoaging, AK, or nevi, especially when other confounding factors, such as age, were considered. In addition, TPY was not associated with melanoma, basal cell carcinoma, or any type of skin cancer. However, ever smokers produced an elevated crude odds ratio (OR=1.99; 95% CI: 1.02–3.88, p=0.043) for squamous cell carcinoma (SCC) compared to non-smokers. In further analysis, TPY of ≤10 produced an elevated multivariable adjusted odds ratio (AOR=4.90; 95% CI: 1.31–18.26, p=0.018) for SCC, but TPY >10 did not (AOR=1.14; 95% CI: 0.22–6.05, p=0.876).

**CONCLUSIONS:**

Smoking was associated, though not dose-dependently, with an increased likelihood of SCC, but it was not associated with basal cell carcinoma or melanoma. However, the impact of smoking on cutaneous photoaging severity, AK, and nevi, appears to be weak.

## INTRODUCTION

Skin cancers have become more common worldwide among White Western populations, and their incidence is rising^1-3^. The aging population, longer life expectancy, and changes in sun-seeking behavior, clothing, and outdoor leisure time habits, are significant influences.

Photoaging (chronic solar damage or photo-induced damage) is the result of prolonged, cumulative exposure to ultraviolet (UV) radiation, which leads to characteristic skin changes. Photodamage produces additional skin changes to normal, intrinsic-type skin aging, such as actinic/solar elastosis and keratosis (AK), skin pigmentation spots, epidermal thickening, and skin wrinkling^4,5^. Skin cancers and their precursors are signs of severe solar UV damage. UV radiation is considered the most significant environmental risk factor for skin cancers^6^. In previous studies, photoaging has been connected to a higher risk of SCC, especially in areas with actinic keratoses (AKs)^7^. Work-related and other UV exposures have been connected to a higher number of AKs, basal cell carcinomas (BCC), and squamous cell carcinomas (SCC)^8^. In addition to being a health hazard, photoaging causes cosmetic and mental harm to an individual. The skin texture and appearance changes caused by photoaging have been described and classified earlier^9,10^.

Tobacco is known to contain multiple carcinogenic substances that increase morbidity^11^. It is one of the leading causes of cancer globally and a causative factor in at least 18 types of cancer^12^. Tobacco smoking is associated with skin wrinkling and thinning of the dermis, resulting in an atrophic and greyish appearance^11^. Smoking is also associated with increased skin elastosis^13^ in both sexes, and with telangiectasia among men^14^. The areas with direct contact with tobacco smoke present with an excess number of wrinkles because the tobacco smoke dries the outermost layer of the skin, stratum corneum, and induces low-grade inflammation^11^. The changes in skin aging correlate with smoked cigarettes per day and pack-years^11^. Therefore, both smoking and UV radiation can have a synergistic influence on skin aging and carcinogenesis.

The relationship between tobacco smoking and skin cancer has been studied previously, but there remains uncertainty about their causality. Predisposing factors in smoking are premature skin aging and stimulation of tumor growth, invasion, and neoangiogenesis^11^. On the other hand, smoking might restrain the inflammation induced by UV radiation and possibly may function as a protective factor in skin cancer development^3^. There is even evidence that smoking has a protective influence on melanomagenesis^3,15,16^. A negative association between all skin cancers and tobacco smoking was found in the UK in 2018, but it was speculated to be a result of bias caused by a high portion of BCC in all skin cancers^17^. In an Australian study, it was found that the association of tobacco smoking with non-melanoma skin carcinomas (NMSC) is conflicting since the risk of BCC was decreased. Still, the risk of SCC was increased among smokers^18^, even though smoking has been shown to increase the risk for SCC, while the connection to BCC is still conflicting^11^. However, there is still a need to clarify the connections between smoking and skin cancers.

Immunosuppression has been connected to increased skin cancer incidence^19-21^. In organ transplant recipients (OTRs), the incidence of SCC is markedly higher than in the general population^19-21^. In a Finnish retrospective study on the effect of solid organ transplantation (SOT) on cancer incidence, 53% of all post-transplant cancers were NMSCs^19^. In addition to increased incidence of SCC, BCC, Kaposi sarcoma, melanoma, and Merkel cell carcinoma, they are expressed in higher numbers among immunocompromised^20^ and SOT patients^19^. In a Swedish national study, over a 100-fold increase in SCC incidence was found among OTRs with a lung or heart transplants or both. However, the association was limited, for the most part, to a small group with several tumors^21^.

Chronic UV exposure and smoking can cause similar changes in skin texture; therefore, the distinction between their individual role in skin aging and carcinogenesis is difficult. Immunosuppression can provide an additional contribution to these events. In this study, the purpose was to assess the association of smoking with cutaneous photoaging, malignancies or pre-malignancies, and pigment cell nevi in subjects at an elevated risk for any type of skin cancer.

## METHODS

### Study subjects

In this cross-sectional study, the subjects were 488 patients; 246 were men (mean age ± SD: 63.9 ± 12.5 years), and 242 were women (60.2 ± 14.2 years). Because multiple skin lesions were studied, a sufficiently large cohort was recruited. The inclusion and exclusion criteria have been described recently in more detail^22,23^. Briefly, the adult subjects aged 18–80 years were evaluated to be at an increased risk for any type of skin cancer as assessed by a dermatologist after reading the referral text or medical records. The subjects were recruited at the outpatient clinic of Kuopio University Hospital, Kuopio, Finland, between May 2017 and October 2020. A flow chart for the recruitment has been presented previously^23^. Factors considered to increase skin cancer risk, for example, were: past or present skin cancer or premalignant lesion, photodamage severity, numerous or atypical pigment cell nevi, skin phototype, family history of melanoma, and/or an immunosuppressive state due to medication for organ transplantation (n=39) or immune-mediated disease (n=55). Subjects with significant psychiatric or neurological disorders affecting markedly the mental health, memory, and/or capability to understand decision-making, convicted prisoners, and pregnant females were excluded from the study. The study was approved by the Ethics Committee of Kuopio University Hospital (71/2017) and it followed the principles of the declaration of Helsinki.

There were 286 subjects with a history of past or present cutaneous malignancy: 100 subjects with melanoma, 202 with BCC, and 38 with SCC. Some subjects had a history of more than one type of cutaneous malignancy. Subjects with only *in situ* melanoma (n=8) were included in the group of all melanomas and any skin cancer (n=286), but were excluded from the group of invasive melanoma (n=92). The subjects with *in situ* SCC (Morbus Bowen) (n=4) were included in the group of SCC and any skin cancer.

### Examination of study subjects

Before the first visit, the subjects filled in a comprehensive data collection form with information on demographic details, body mass index (BMI), different aspects of UV exposure, diseases in skin and other organs, medication, and smoking^22,23^. In this study, tobacco smoking was defined as the use of combustible forms of tobacco, such as cigarettes, cigars, cigarillos, or tobacco pipes. Cumulative use of tobacco products was calculated as tobacco pack-years (TPY), defined as the average number of smoked cigarettes per day divided by a pack of cigarettes (20 sticks) and then multiplied by years of smoking. Smoked years were calculated with the following questions: ‘If you have ever smoked, at which age did you start?’ and ‘If you have stopped smoking, how long ago did you stop it?’. The number of smoked cigarettes per day was calculated as an average number from answer options: 1–2, 3–10, 11–20, and >20 cigarettes, cigars or tobacco pipes. Those who chose the option ‘occasionally, but not regularly’, were considered non-smokers. Upon entry, a study dermatologist checked all medical records and thoroughly examined the subject’s skin by paying particular attention to photoaging, premalignant and malignant lesions, and pigment cell nevi. Before the recruitment of subjects, the three dermatologists of the study were trained to evaluate skin lesions, photoaging level, and nevus number, to ensure equal assessment.

PhotoAging Area and Severity Index (PAASI) is a score for evaluating the photoaging level in all skin areas of the head, torso, and upper and lower limbs^23^. The level of photoaging at each skin site was evaluated with the following scores: 0 = no marked solar damage (intrinsic skin aging), 1 = mild damage, 2 = moderate damage, 3 = severe damage with AK, 4 = very severe damage with several AKs. The PAASI score ranged 0–400.

Pigment cell nevi were counted, and subjects were divided into subgroups of 0–20, 21–50, 51–100, and >100 nevi as described^23^. Actinic keratoses were counted similarly, and subjects were divided into subgroups of 0, 1, 2, 3, 4–10, or >10 AKs ^23^. If a diagnostic uncertainty was encountered, a biopsy was taken.

### Blood sample

A blood sample was taken from each subject and analyzed in the hospital laboratory of Kuopio University Hospital for the levels of hemoglobin (Hb), thrombocytes, and white blood cell count and differential.

### Statistical analysis

Statistical analysis was performed using SPSS Statistics version 27. In the case of continuous variables, Levene’s test was performed to assess the equality of variance, after which a 2-tailed t-test or one-way ANOVA was computed. In the case of non-parametric variables, the Mann-Whitney test was used, and the chi-squared test was performed in categorical variables. Spearman’s rank correlation coefficients were used to evaluate correlation levels between variables. Crude and multivariable logistic regression analyses with their 95% confidence intervals (CI) were conducted to determine the factors associated with nevi, skin cancers, and photoaging. Multivariable models were formed by including variables with statistical significance in preceding analyses and also by including clinically relevant variables previously known to be associated with skin cancers. Results were considered statistically significant for p<0.05.

## RESULTS

### Correlation between tobacco pack-years and skin photodamage indicators or mole count in 488 subjects with competent or compromised immune system

The results are summarized in Table 1. The mean age and BMI of male and female subjects with immunosuppression (IS) or non-IS, were similar in each group. Even though there were some significant differences in blood cell parameters between IS and non-IS subjects, the values were within normal limits. The TPY value did not differ between IS and non-IS subjects in either gender, though male subjects expectedly showed higher TPY than females regardless of the immune status. The result was similar when comparing TPY solely in subjects with past or present smoking history and excluding those without any smoking history.

**Table 1:** Correlation between tobacco pack-years and other parameters in 488 subjects with compromised (IS) or competent (non-IS) immune system at Kuopio University Hospital between May 2017 and October 2020

	*All (N=488) Mean ± SD Sc Sig*	*Non-IS (N=394) Mean ± SD Sc Sig*	*IS (N=94) Mean ± SD Sc Sig*	*Males (N=246) Mean ± SD Sc Sig*	*non-IS (N=195) Mean ± SD Sc Sig*	*IS (N=51) Mean ± SD Sc Sig*	*Females (N=242) Mean ± SD Sc Sig*	*Non-IS (N=199) Mean ± SD Sc Sig*	*IS (N=43) Mean ± SD Sc Sig*	*IS vs non-IS p (All) p (Males) p (Females)*
TPY	5.0 ± 11.3	4.9 ± 11.8	5.6 ± 8.8	7.6 ± 13.9	7.5 ± 14.7	8.3 ± 10.1	2.4 ± 7.0	2.4 ± 7.2	2.4 ± 5.5	0.616 0.715 0.967
Age (years)	62.1 ± 13.5 0.134 0.003	62.5 ± 13.5 0.142 0.005	60.3 ± 12.9 0.168 0.106	63.9 ± 12.5 0.169 0.008	64.7 ± 12.5 0.189 0.008	61.2 ± 11.7 0.222 0.118	60.2 ± 14.2 0.015 0.821	60.5 ± 14.2 -0.004 0.952	59.1 ± 14.2 0.107 0.494	0.139 0.070 0.582
BMI (kg/m^2^)	26.7 ± 4.8 0.094 0.038	26.9 ± 4.8 0.052 0.300	25.8 ± 4.8 0.317 0.002	26.7 ± 4.0 0.005 0.936	26.9 ± 3.9 -0.057 0.431	26.0 ± 4.3 0.319 0.023	26.7 ± 5.5 0.187 0.004	27.0 ± 5.5 0.169 0.017	25.6 ± 5.4 0.278 0.071	0.045 0.163 0.142
PAASI score	67.0 ± 44.0 0.179 <0.001	68.0 ± 44.0 0.176 <0.001	64.0 ± 44.0 0.210 0.044	72.0 ± 43.0 0.191 0.003	73.0 ± 43.0 0.201 0.005	66.0 ± 42.0 0.179 0.218	62.0 ± 45.0 0.115 0.074	63.0 ± 45.0 0.091 0.201	61.0 ± 47.0 0.228 0.141	0.464 0.345 0.820
AK count	2.3 ± 1.8 0.113 0.012	2.4 ± 1.8 0.156 0.002	2.0 ± 1.6 0.006 0.956	2.8 ± 2.0 0.091 0.156	2.9 ± 2.0 0.167 0.019	2.4 ± 1.9 -0.114 0.427	1.9 ± 1.5 -0.002 0.981	1.9 ± 1.5 0.009 0.899	1.5 ± 1.1 -0.021 0.892	0.015 0.067 0.039
Mole count	1.9 ± 1.1 -0.130 0.004	2.0 ± 1.1 -0.136 0.007	1.6 ± 0.9 -0.056 0.592	1.9 ± 1.1 -0.232 <0.001	2.0 ± 1.1 -0.235 <0.001	1.7 ± 0.9 -0.171 0.234	1.9 ± 1.1 -0.037 0.569	2.0 ± 1.1 -0.033 0.646	1.5 ± 0.9 -0.064 0.686	0.002 0.149 0.003
Leukocyte cell count (×10^9^/L)	6.3 ± 1.7 0.099 0.030	6.2 ± 1.7 0.127 0.012	6.5 ± 2.0 0.005 0.962	6.3 ± 1.7 0.109 0.087	6.2 ± 1.7 0.158 0.027	6.7 ± 2.0 -0.046 0.749	6.3 ± 1.7 0.109 0.093	6.3 ± 1.6 0.130 0.070	6.4 ± 2.0 0.000 0.999	0.121 0.084 0.700
Neutrophil cell count (×10^9^/L)	4.0 ± 3.3 0.017 0.709	3.9 ± 3.6 0.032 0.531	4.3 ± 1.9 -0.049 0.646	3.9 ± 1.5 0.027 0.680	3.7 ± 1.3 0.055 0.448	4.4 ± 1.9 -0.057 0.692	4.1 ± 4.5 0.009 0.892	4.0 ± 4.9 0.023 0.746	4.1 ± 1.8 -0.084 0.604	0.275 0.001 0.921
Lymphocyte cell count (×10^9^/L)	1.9 ± 0.7 0.110 0.016	1.9 ± 0.7 0.133 0.009	1.7 ± 0.7 0.066 0.531	1.8 ± 0.7 0.176 0.006	1.8 ± 0.7 0.232 0.002	1.6 ± 0.7 0.047 0.744	1.9 ± 0.7 0.117 0.075	2.0 ± 0.7 0.123 0.088	1.7 ± 0.7 0.095 0.555	0.002 0.056 0.016
Basophil cell count (×10^9^/L)	0.044 ± 0.052 0.037 0.425	0.047 ± 0.052 0.043 0.401	0.036 ± 0.048 0.041 0.700	0.044 ± 0.050 0.000 0.997	0.045 ± 0.050 0.040 0.587	0.041 ± 0.050 -0.098 0.504	0.044 ± 0.054 0.095 0.147	0.048 ± 0.055 0.062 0.394	0.029 ± 0.046 0.256 0.106	0.069 0.586 0.045
Monocyte cell count (×10^9^/L)	0.39 ± 0.14 0.188 <0.001	0.39 ± 0.13 0.195 <0.001	0.40 ± 0.16 0.176 0.093	0.42 ± 0.15 0.167 0.010	0.42 ± 0.15 0.157 0.031	0.42 ± 0.18 0.224 0.114	0.36 ± 0.11 0.133 0.042	0.36 ± 0.11 0.165 0.022	0.37 ± 0.11 -0.020 0.902	0.398 0.874 0.403
Eosinophil cell count (×10^9^/L)	0.18 ± 0.18 0.047 0.309	0.18 ± 0.15 0.027 0.606	0.17 ± 0.2 0.182 0.084	0.18 ± 0.12 0.056 0.395	0.18 ± 0.13 0.025 0.738	0.15 ± 0.09 0.232 0.106	0.18 ± 0.23 -0.003 0.961	0.18 ± 0.17 -0.013 0.857	0.20 ± 0.41 0.075 0.643	0.610 0.101 0.704
Hemoglobin (g/L)	141.0 ± 12.0 0.156 <0.001	142.0 ± 12.0 0.135 0.007	139.0 ± 13.0 0.261 0.011	147.0 ± 12.0 -0.036 0.574	148.0 ± 11.0 -0.075 0.298	144.0 ± 14.0 0.110 0.442	136.0 ± 9.0 0.081 0.213	136.0 ± 9.0 0.038 0.600	133.0 ± 9.0 0.273 0.076	0.022 0.015 0.061
Thrombocyte cell count (×10^9^/L)	246.0 ± 59.0 -0.006 0.894	243.0 ± 57.0 0.010 0.841	256.0 ± 70.0 -0.096 0.357	230.0 ± 53.0 0.053 0.412	226.0 ± 49.0 0.093 0.198	243.0 ± 63.0 -0.118 0.409	262.0 ± 62.0 0.121 0.061	260.0 ± 58.0 0.112 0.117	272.0 ± 77.0 0.155 0.321	0.057 0.040 0.242

Sc: Spearman correlation coefficient. Sig: significance. TPY: tobacco pack-years. BMI: body mass index: PAASI: PhotoAging Area and Severity Index. AK: actinic keratosis. IS: immunosuppression. The differences between continuous variables were tested with the 2-tailed t-test for which the equality of variance was evaluated by the p-value of the Levene test. The differences between the non-parametric variables (AK and mole count) were tested with the Mann-Whitney test.

TPY value correlated positively with age in all and non-IS subjects, but this slight correlation was confined to male subjects only (Table 1). Concerning BMI, a positive correlation was seen in all and IS subjects, male IS subjects, as well as in all female and non-IS subjects. Similar to age, TPY correlated positively with PAASI in all subjects, non-IS, and IS subjects, but this correlation was related to male subjects only. In agreement with this result, TPY correlated positively with AK count in all and non-IS subjects, but this relation was confined to male non-IS subjects. In contrast, the correlation between TPY and mole count was inverse in all and non-IS subjects, significantly so in male subjects but not significantly in female ones.

A significantly positive correlation was observed between TPY and several blood parameters, such as Hb, leukocytes, monocytes, or lymphocytes, in all non-IS subjects (Table 1). These white blood cell parameters were confined to non-IS males, though, in the case of monocytes, to non-IS females, too.

### Past or present skin malignancies and pigment cell nevi in smokers and non-smokers

To study the dose-response effect of smoking and to obtain a sufficient number of cases in 2 smoking groups, the subjects with a history of smoking were divided into two groups, ≤10 TPY and >10 TPY (Table 2). The male/female ratio, age, PAASI, and Fitzpatrick phototype increased with increasing TPY. Still, there was no difference in lifetime sun exposure and sunburns, main working environment, solarium, UV treatment, nevus count, immunosuppression, OTR or facial photoaging score. There was no difference between smoking groups concerning past or present melanoma, BCC, SCC, or any skin cancer either. However, significant, but not dose-dependent, changes were seen in AKs.

**Table 2:** Past or present skin malignancies and nevi in smokers and non-smokers in 488 subjects at Kuopio University Hospital between May 2017 and October 2020

	*Never smoker N=330*	*≤10 TPY N=69*	*>10 TPY N=89*	p*
Age (years), mean ± SD	60.6 ± 14.3	63.8 ± 13.2	65.4 ± 9.5	0.005 ANOVA
	***n (%)***	***n (%)***	***n (%)***	
**Gender**				<0.001
Male	136 (41.2)	46 (66.7)	64 (71.9)	
Female	194 (58.8)	23 (33.3)	25 (28.1)	
**BMI** (kg/m^2^)	N=330	N=69	N=88	0.241 ANOVA
mean ± SD	26.4 ± 4.9	27.4 ± 5.1	26.8 ± 3.9	
	N=330	N=69	N=89	
	***n (%)***	***n (%)***	***n (%)***	
**Immunosuppression**	57 (17.3)	12 (17.4)	25 (28.1)	0.065
**OTR**	26 (7.9)	4 (5.8)	10 (11.2)	0.435
**Invasive melanoma**	66 (20.0)	11 (15.9)	15 (16.9)	0.638
**All melanomas**	73 (22.1)	12 (17.4)	15 (16.9)	0.434
**Basal cell carcinoma**	128 (38.8)	30 (43.5)	44 (49.4)	0.181
**Squamous cell carcinoma**	20 (6.1)	9 (13.0)	9 (10.1)	0.096
**Any skin cancer**	188 (57.0)	38 (55.1)	60 (67.4)	0.168
	***%***	***%***	***%***	
**Number of present AKs**				0.015
0	59.7	40.6	55.1	
1	13.0	10.1	11.2	
2	4.2	11.6	3.4	
3	6.4	5.8	4.5	
4–10	9.4	23.2	14.6	
>10	7.3	8.7	11.2	
	***n (%)***	***n (%)***	***n (%)***	
**Subjects with at least one AK**	133 (40.3)	41 (59.4)	40 (44.9)	0.014
**PAASI score**	N=328	N=69	N=88	0.003 ANOVA
mean ± SD	62.23 ± 44.07	72.86 ± 42.15	79.24 ± 43.15	
	N=330	N=69	N=88	
	***%***	***%***	***%***	
**Facial photoaging score**				0.108
0	4.2	2.9	0.0	
1	24.8	23.2	20.2	
2	45.8	36.2	42.7	
3	23.9	36.2	37.1	
4	1.2	1.4	0.0	
**Facial photoaging score**	N=330	N=69	N=89	0.021
0–2 vs 3–4	247 vs 83	43 vs 26	56 vs 33	
**Mole count**	N=328	N=69	N=89	0.054
0–50 vs >50	227 vs 101	51 vs 18	73 vs 16	
	***%***	***%***	***%***	
**Skin phototype** (Fitzpatrick)	N=315	N=63	N=85	0.048
1	6.7	1.6	2.4	
2	42.5	46.0	34.1	
3	46.7	52.4	55.3	
4	4.1	0.0	8.2	
**Fitzpatrick score**	N=329	N=69	N=88	0.037 ANOVA
mean ± SD	14.15 ± 4.47	14.25 ± 4.58	15.55 ± 4.83	
	***%***	***%***	***%***	
**Lifetime sun exposure**	N=325	N=68	N=89	0.266
Very seldom	20.9	10.3	16.9	
Occasionally	38.5	39.7	33.7	
Often	27.4	35.3	29.2	
Very often	13.2	14.7	20.2	
**Lifetime sunburns**	N=329	N=68	N=89	0.290
Seldom	31.0	35.3	32.6	
Occasionally	49.8	39.7	40.4	
Often	19.1	25.0	27.0	
**Solarium**	N=328	N=69	N=89	0.138
Never	68.0	75.4	76.4	
0–30	26.8	17.4	15.7	
31–100	5.2	7.2	7.9	
**UV light treatment**	N=313	N=67	N=85	0.666
Never	89.8	92.5	94.1	
0–30	6.7	6.0	4.7	
31–100	3.5	1.5	1.2	
**Main working environment**	N=328	N=67	N=89	0.094
Outdoors	5.8	4.5	11.2	
Indoors	72.0	62.7	61.8	
Both variably	22.3	32.8	27.0	

TPY: tobacco pack-years. BMI: body mass index. OTR: organ transplant recipients. AKs: actinic keratoses. PAASI: PhotoAging Area and Severity Index. UV: ultraviolet.

* Chi-squared test.

By comparing the subjects with >10 TPY to never smokers, there was a significantly higher percentage of subjects with male gender, IS, more severe facial photoaging score, subjects with a low number of moles, and a higher PAASI score among smokers than non-smokers. No significant difference was seen in other variables.

### Binary logistic regression analysis

Both crude and multivariable logistic regression analyses were used to evaluate the factors associated with endpoint variables. The results are shown in detail in Table 3 and in Supplementary file Tables S1–S7.

**Table 3:** The logistic regression analysis and consequent odds ratios for subjects with a history of squamous cell carcinoma compared to control subjects without squamous cell carcinoma in 488 subjects at Kuopio University Hospital between May 2017 and October 2020

*Variable*	*OR*	*95% CI*	*p*	*AOR*	*95% CI*	*p*
**Pack-years**						
Never smoker ®	1			1		
≤10 TPY	2.33	1.101–5.353	0.047	4.90	1.313–18.261	0.018
>10 TPY	1.74	0.765–3.976	0.186	1.14	0.216–6.051	0.876
**PAASI score**	1.02	1.009–1.023	<0.001	1.02	1.006–1.034	0.004
**Age** (years)	1.12	1.064–1.185	<0.001	1.13	1.038–1.234	0.005
**Gender**						
Male ®	1			1		
Female	0.57	0.289–1.136	0.111	2.33	0.522–10.430	0.267
**BMI**	1.07	1.010–1.132	0.022	1.06	0.965–1.169	0.217
**Lifetime sun exposure**						
Very seldom ®	1			1		
Occasionally	1.63	0.518–5.161	0.402	2.10	0.325–13.518	0.436
Often	1.61	0.490–5.298	0.432	1.12	0.155–8.106	0.911
Very often	3.80	1.156–12.476	0.028	7.22	0.846–61.590	0.071
**Main working environment**						
Outdoors ®	1			1		
Indoors	0.26	0.093–0.699	0.008	0.25	0.039–1.595	0.142
Both variably	0.79	0.284–2.184	0.646	0.52	0.086–3.168	0.481
**Lifetime sunburns**						
Seldom ®	1			1		
Occasionally	1.35	0.614–2.965	0.457	9.31	2.005–43.207	0.004
Often	1.14	0.434–2.981	0.794	5.48	0.773–38.889	0.089
**Solarium**						
Never ®	1			1		
0–30	0.45	0.172–1.192	0.109	0.25	0.053–1.215	0.086
31–100	0.00	0.000	0.998	0.00	0.000	0.998
**UV light treatment**	1			1		
Never ®						
0–30	1.86	0.613–5.648	0.273	0.25	0.020–3.048	0.274
31–100	0.00	0.000	0.999	0.00	0.000	0.998
**Skin phototype** (Fitzpatrick)	1			1		
1 ®						
2	1.60x10^8^	0.000	0.998	1.98x10^9^	0.000	0.998
3	0.58x10^8^	0.000	0.998	7.23x10^8^	0.000	0.998
4	5.38x10^8^	0.000	0.998	1.32x10^10^	0.000	0.997
**Immunosuppression**						
Non-IS ®	1			1		
IS	1.14	0.506–2.578	0.748	1.40	0.312–6.312	0.659
**Hemoglobin count**	1.02	0.995–1.049	0.118	1.08	1.019–1.140	0.009
**Leukocyte count**	1.17	0.983–1.399	0.077	164.05	0.069–391020.029	0.199
**Monocyte count**	6.77	0.783–58.595	0.082	0.00	0.000–1.143	0.053
**Lymphocyte count**	1.32	0.864–2.004	0.201	0.01	0.000–29.068	0.273
**Neutrophile count**	1.01	0.933–1.094	0.798	0.01	0.000–22.497	0.240
**Basophile count**	2.93	0.005–1639.383	0.739	0.00	0.000–2.785	0.073
**Eosinophile count**	1.59	0.377–6.670	0.529	0.01	0.000–34.438	0.266

AOR: adjusted odds ratio; in the multivariable analysis, all variables were simultaneously present in the analysis. TPY: tobacco pack-years. PAASI: PhotoAging Area and Severity Index. BMI: body mass index. UV: ultraviolet. IS: immunosuppression. ® Reference categories.

In the case of a history of any skin cancer (Supplementary file Table S1), an elevated crude OR was found for PAASI, age, and lifetime sunburn history, but a decreased one for immunosuppression. In multivariable analysis, an elevated AOR was found for the age and highest level of lifetime sun exposure but a decreased one for immunosuppression. Concerning the endpoint variable of a history of BCC (Supplementary file Table S2), an elevated crude OR was found for PAASI and age but a decreased one for immunosuppression. In multivariable analysis, an elevated AOR was seen for the age, highest level of lifetime sun exposure, and UV-light treatment for 0–30 times. There was no relation to smoking history.

In the case of the endpoint variable of a history of SCC, ever smokers produced an elevated crude odds ratio (OR=1.99; 95% CI: 1.02–3.88, p=0.043) compared to never smokers. In further analysis (Table 3), an elevated crude OR was found for ≤10 TPY (OR=2.33; 95% CI: 1.10–5.35, p=0.047), PAASI, age, BMI, and the highest level of lifetime sun exposure, but a decreased one for indoor working environment. In multivariable analyses, an elevated AOR was found for ≤10 TPY (AOR=4.90; 95% CI: 1.31–18.26, p=0.018), PAASI, age, lifetime sunburn history, and Hb. To highlight this finding, elevated odds ratios were found in both simple and multivariable analysis for ≤10 TPY, but not for >10 TPY.

With respect to the endpoint variable of a history of melanoma (Supplementary file Table S3), an elevated crude OR was found for mole counts of 21–50, 51–100, and >100, as well as for the lifetime sunburn history, but a decreased likelihood for skin phototype and immunosuppression. In multivariable analysis, an elevated AOR was found for mole counts of 21–50, 51–100, and >100, but a decreased one for immunosuppression. Nonetheless, no relation to smoking was found.

Smoking may affect the entire skin and its PAASI. Therefore, the subjects with PAASI higher than the median were compared to controls with PAASI below or equal to the median (Supplementary file Table S4). An elevated crude OR was found for the age, female gender, highest level of lifetime sun exposure, and monocyte count, but decreased one for female gender, indoor working environment, and occasional lifetime sunburns. In multivariable analysis, only the age and highest level of lifetime sun exposure produced an elevated AOR, but BMI a decreased one. Smoking was not significantly related to PAASI. Concerning facial photoaging, the photodamage score of 3–4 was compared to 0–2 (Supplementary file Table S5). An elevated crude OR was found for smoking >10 TPY and ≤10 TPY when compared to never smokers. Also, the age, leukocyte count, and monocyte count revealed an elevated crude OR. A decreased crude OR was found for the female gender and solarium use for 0–30 times. In multivariable analysis, an elevated AOR was observed only for age but not for smoking.

Concerning the endpoint variable and marker of carcinogenesis, AK (Supplementary file Table S6), an elevated crude OR was found for smoking ≤10 TPY, age, UV light treatment for 0–30 times, BMI, leukocyte count, and monocyte count, but a decreased one for the female gender, indoor working environment, occasional lifetime sunburns, immunosuppression, and leukocyte count. However, an elevated AOR was found only for age in multivariable analysis.

In the case of pigment cell nevi, subjects with >50 moles were compared to control subjects with ≤50 moles (Supplementary file Table S7). In simple logistic regression analysis, a decreased OR was found for smoking >10 TPY, PAASI, age, immunosuppression, leukocyte count, and monocyte count. A history of occasional lifetime sunburns revealed an elevated crude OR. In multivariable analysis, age, immunosuppression, and a history of occasional lifetime sun exposure were related to a decreased AOR, whereas for BMI it was elevated. However, there was no association with smoking.

## DISCUSSION

The primary purpose of this cross-sectional study on 488 subjects was to determine whether there is an association between tobacco smoking and cutaneous photoaging, AKs, cancers, or nevi in subjects considered to have an elevated risk for any type of skin cancer.

As expected, the TPY value was higher in males than females, but there was no difference between immunocompetent and immunocompromised subjects in either gender. In the Spearman correlation analysis, the markers of photoaging and carcinogenesis, PAASI and AK, showed a significant positive correlation with TPY in all subjects. Still, it was confined to males, especially to immunocompetent ones. However, the age of subjects revealed a similar significant positive correlation to TPY in all subjects, but it was confined to immunocompetent males again. This suggests that age is the predominant factor for PAASI and AK. There was an unexpected finding on the positive correlation between BMI and TPY in all subjects, which was related to immunocompromised males and immunocompetent females. Even though smoking has been associated with lower body weight, a possibility of the positive correlation may be that these subjects had attempted to lower body weight by smoking^24,25^. Alternatively, the observed positive correlation between BMI and TPY might be a result of different distributions of heavy or former smokers in each smoking group^26^. Another finding was the positive correlation between TPY and Hb, leukocytes, lymphocytes, or monocytes, especially among immunocompetent males. However, similar associations have also been recognized previously, e.g. in a large Danish study on 104607 subjects^27^. In the case of immunocompromised subjects, there was no correlation between TPY and white blood cells. An explanation may be that the immunosuppressive medication has an interfering effect on this relationship.

When the subjects were divided into three groups according to non-smoking or smoking more or less than 10 TPY, the results on the markers of photoaging and carcinogenesis were similar. That is, PAASI, facial photoaging, and AKs increased together with the increase in TPY, but so did the male/female ratio, age, and Fitzpatrick skin type. To clarify the significance of TPY in facial photoaging, PAASI, and AK, logistic regression analysis was utilized. Even though significant simple ORs were found for TPY, these significances disappeared in multivariable analysis, leaving age as the only essential factor. Therefore, the association of smoking with facial photoaging, PAASI, and AK appears to be weak at most. In a recent multinational cross-sectional study, both current and former smokers were found to express more advanced signs of skin aging compared to never smokers in several facial features associated with aging^28^, and a cumulative effect of smoking in aging skin was suggested.

There was no statistically significant association between smoking groups and a history of melanoma, BCC, SCC, or any skin cancer. Since skin cancers have shown associations with smoking in previous literature, the relationship between smoking and any skin cancer, BCC, SCC, and melanoma was tested with the logistic regression analysis, too. There was no relationship between smoking and any skin cancer, BCC, or melanoma in this analysis. In the case of SCC, a significant OR was found in both simple and multivariable analysis for smoking of less than 10 TPY, thus suggesting an increased risk for SCC among smokers. However, the age and PAASI produced increased ORs as well. Instead, there was no such increase in OR for SCC in subjects with smoking of over 10 TPY. One possibility for this disparity is that smoking and nicotine at sufficiently high levels may have protective or suppressive effects on skin inflammation and, thereby, on carcinogenesis^3,29^. Alternatively, this is a coincidence due to the number of SCC cases. The present findings on SCC and BCC are similar to previous studies’ findings. In the Australian population-based cohort study, it was shown that current smokers had a significantly lower risk for BCC but a higher risk for SCC compared to never smokers. In contrast, former and never smokers shared similar risks for both keratinocyte carcinomas. However, a detection bias was considered to possibly affect the results^18^. In a study in Bosnia Herzegovina, an analysis of 131 participants revealed no significant association between smoking and BCC but rather a possible inverse relationship^30^. A systematic review of the impact of different lifestyle factors on NMSC found no associations between BCC and smoking but a significant 52% increase in the risk of SCC^31^, which is quite similar to the 99.3% increase in the present study.

In contrast to SCC, the risk of malignant melanoma has previously been reported to decrease among male smokers but not among female ones^3^. In a large cohort of Swedish male construction workers, evidence was found for a decreased risk of malignant and *in situ* melanoma by tobacco smoking and snuff using^15^. In a US prospective cohort study on females, it was reported that in current female smokers, there was a lower risk for malignant melanoma, though the same finding was not revealed in former smokers^16^. Nevertheless, a recent study on Finnish subjects showed that smoking is an independent marker of poor prognosis in cutaneous melanoma^32^. In addition, smoking has been reported to be an independent risk factor for cutaneous melanoma in the elderly (age ≥ 60 years)^33^. The age range in the present study was wide, with a mean age of slightly over 60 years, which might explain the result that there was no relationship between smoking and melanoma. However, age or gender produced no significant ORs in the logistic regression analysis.

There is little research about the relationship between smoking and nevi. An Austrian study found no associations between smoking and nevus count, atypical nevi, or lentigines^34^. The Spearman correlation analysis revealed a negative correlation between nevus count and TPY in all immunocompetent male subjects in this study. It is possible that nevi are related to age, PAASI, and AK because these changes were confined to the same subject groups. However, in the multivariable logistic regression analysis, smoking was not associated with nevi, even though in the simple analysis it was, with a decreased OR. Therefore, the association of smoking with nevi appears to be minimal.

Immunosuppression, such as that in OTRs, has been connected to increased incidence of a variety of skin cancer types^20^. In addition, there are signs of increased nevus count among IS patients^35^, although there are only a few studies on that topic in the literature. Also, eruptive melanocytic nevi, characterized by suddenly appearing multiple nevi, has shown some associations with several immunosuppressive medications^36^.

In this study, immunosuppression was presented as a protective factor in the simple and in the multivariable logistic regression analyses on any skin cancer, melanoma, and mole count, but nevi as a significant risk factor of melanoma. In the case of AK, the protective association was found only in the simple or crude analysis. However, this unexpected result may be due to selection bias of study subjects as well as to the fact that the immunocompromised cohort consisted of subjects with a heterogeneous disease background^30^. Nevertheless, the subjects with immunosuppression showed some differences compared to immunocompetent ones, such as no significant correlation between TPY and AK or nevus count.

### Strengths and limitations

The strength of this study is that all skin sites were thoroughly examined by experienced dermatologists. A weakness is the cohort-based study population, which does not represent the general population. Thus, a selection bias is the risk in this research design. In addition, bias may be caused by the fact that the answers on past smoking are based on the recall of relatively aged subjects in the study. Furthermore, the study design cannot show causality between smoking and SCC. Of note is also the fact that the subjects were dichotomized into two groups, i.e. subjects with or without a history of skin cancer, yet the control group could contain subjects with another type of skin cancer.

## CONCLUSIONS

The essential finding of this study is that there was a significant relationship between smoking of less than 10 TPY and a history of SCC, but not so between smoking of over 10 TPY and SCC. Therefore, the dose dependence for SCC risk is lacking. Nevertheless, a 99.3 % increase in the risk of SCC was found among ever smokers. In contrast to SCC, smoking was not significantly associated with BCC, melanoma, skin cancer, AKs, photoaging severity, and pigment cell nevi, especially when other potential confounding factors, such as age, were taken into consideration. Therefore, the impact of smoking on cutaneous photoaging and carcinogenesis appears to be weak, and we identified within our study SCC as the only skin malignancy with some connection to smoking.

## Supplementary Material



## Data Availability

The data supporting this research are available from the authors on reasonable request.
