# Supplementary material for: Tobacco smoking is associated with cutaneous squamous cell carcinoma but not with basal cell carcinoma or melanoma in adult subjects at risk of skin cancer: A cross-sectional study
Source: Tob Induc Dis. 2024 May 14;22:10.18332/tid/185299. doi: 10.18332/tid/185299 (PMC11091865; doi:10.18332/tid/185299)
Supplement: Supplementary file 1 [file TID-22-76-s1.pdf]

Supplementary table S1. The logistic regression analysis and consequent Odds Ratios for subjects with any skin cancer compared to control subjects without skin cancer in 488 subjects at Kuopio University Hospital between May 2017 and October 2020.

Variable	Simple Odds Ratio	95% Confidence interval	P value	Multivariable Odds Ratio	95% Confidence interval	P value
Pack years:						
Never smoker	Ref., 1			Ref., 1		
≤10 TPY	0.93	0.549-1.560	0.772	0.93	0.478-1.799	0.824
>10 TPY	1.56	0.954-2.561	0.076	1.21	0.660-2.234	0.533
PAASI score	1.01	1.007-1.016	<.001	1.00	0.995-1.008	0.618
Age	1.05	1.034-1.065	<.001	1.05	1.026-1.070	<.001
Gender:						
Male	Ref., 1			Ref., 1		
Female	0.95	0.662-1.349	0.754	0.89	0.502-1.591	0.703
BMI	1.02	0.984-1.062	0.263	1.01	0.959-1.053	0.840
Lifetime sun exposure:						
Very seldom	Ref., 1			Ref., 1		
Occasionally	1.06	0.642-1.760	0.812	1.22	0.647-2.309	0.537
Often	1.27	0.745-2.152	0.384	1.19	0.580-2.440	0.637
Very often	1.82	0.956-3.457	0.068	2.56	0.997-6.550	0.051
Main working environment:						
Outdoors	Ref., 1			Ref., 1		
Indoors	0.68	0.329-1.394	0.290	1.01	0.385-2.622	0.992
Both variably	0.68	0.313-1.483	0.334	0.87	0.322-2.363	0.788
Lifetime sunburns:						
Seldom	Ref., 1			Ref., 1		
Occasionally	1.03	0.684-1.547	0.894	1.27	0.755-2.120	0.372
Often	1.96	1.164-3.287	0.011	1.70	0.813-3.549	0.158
Solarium:						
Never	Ref., 1			Ref., 1		
0-30	1.11	0.721-1.706	0.638	1.09	0.619-1.907	0.772
31-100	0.52	0.244-1.100	0.087	0.41	0.149-1.149	0.090
UV light treatment:						
Never	Ref., 1			Ref., 1		
0-30	1.78	0.802-3.961	0.156	1.88	0.687-5.130	0.219
31-100	0.73	0.251-2.115	0.561	0.66	0.186-2.310	0.511
Skin phototype (Fitzpatrick):						
1	Ref., 1			Ref., 1		
2	0.80	0.326-1.955	0.623	1.63	0.554-4.784	0.375
3	0.58	0.239-1.407	0.228	1.31	0.430-3.982	0.636
4	0.93	0.266-3.244	0.908	1.56	0.327-7.479	0.575
Immunosuppression:						
Non-IS	Ref., 1			Ref., 1		
IS	0.44	0.280-0.691	<.001	0.42	0.233-0.753	0.004
Hemoglobin count	1.00	0.988-1.018	0.699	1.00	0.976-1.019	0.820
Leukocyte count	1.04	0.938-1.156	0.448	8.14	0.375-176.439	0.182
Monocyte count	1.25	0.334-4.694	0.739	0.04	0.001-1.267	0.068
Lymphocyte count	1.21	0.923-1.584	0.167	0.16	0.007-3.588	0.247
Neutrophile count	0.97	0.902-1.038	0.356	0.13	0.006-2.812	0.192
Basophile count	2.30	0.068-77.985	0.643	0.29	0.002-38.152	0.616
Eosinophile count	1.19	0.421-3.378	0.741	0.14	0.005-4.104	0.256

In multivariable analysis, all variables were simultaneously present in the analysis.
TPY, tobacco pack years; PAASI, PhotoAging Area and Severity Index; BMI, body mass index; UV, ultraviolet; IS, immunosuppression; Ref., reference.

Supplementary table S2. The logistic regression analysis and consequent Odds Ratios for subjects with a history of basal cell carcinoma compared to control subjects without basal cell carcinoma in 488 subjects at Kuopio University Hospital between May 2017 and October 2020.

Variable	Simple Odds Ratio	95% Confidence interval	P value	Multivariable Odds Ratio	95% Confidence interval	P value
Pack years:						
Never smoker	Ref., 1			Ref., 1		
≤10 TPY	1.21	0.718-2.052	0.469	1.27	0.656-2.475	0.474
>10 TPY	1.54	0.964-2.471	0.071	1.10	0.608-2.007	0.745
PAASI score	1.01	1.005-1.014	<.001	1.00	0.995-1.007	0.762
Age	1.06	1.038-1.073	<.001	1.06	1.035-1.084	<.001
Gender:						
Male	Ref., 1			Ref., 1		
Female	0.91	0.641-1.303	0.619	0.70	0.394-1.258	0.236
BMI	1.00	0.959-1.033	0.807	0.97	0.922-1.021	0.249
Lifetime sun exposure:						
Very seldom	Ref., 1			Ref., 1		
Occasionally	1.06	0.631-1.776	0.828	1.32	0.678-2.570	0.415
Often	1.40	0.820-2.394	0.218	1.65	0.790-3.466	0.182
Very often	1.68	0.899-3.126	0.104	3.13	1.240-7.920	0.016
Main working environment:						
Outdoors	Ref., 1			Ref., 1		
Indoors	1.07	0.534-2.124	0.859	2.00	0.769-5.197	0.156
Both variably	1.07	0.505-2.260	0.862	1.58	0.588-4.254	0.364
Lifetime sunburns:						
Seldom	Ref., 1			Ref., 1		
Occasionally	0.77	0.507-1.157	0.205	0.87	0.512-1.468	0.595
Often	1.23	0.756-2.014	0.401	1.03	0.498-2.135	0.935
Solarium:						
Never	Ref., 1			Ref., 1		
0-30	1.07	0.699-1.629	0.764	0.95	0.542-1.659	0.851
31-100	0.50	0.214-1.141	0.099	0.48	0.161-1.419	0.184
UV light treatment:						
Never	Ref., 1			Ref., 1		
0-30	2.09	0.998-4.373	0.051	2.99	1.168-7.651	0.022
31-100	0.84	0.276-2.543	0.755	2.00	0.308-3.911	0.886
Skin phototype (Fitzpatrick):						
1	Ref., 1			Ref., 1		
2	0.86	0.366-2.004	0.720	0.75	0.259-2.152	0.588
3	0.80	0.342-1.854	0.598	0.71	0.237-2.140	0.545
4	0.79	0.237-2.621	0.697	0.46	0.096-2.143	0.319
Immunosuppression:						
Non-IS	Ref., 1			Ref., 1		
IS	0.61	0.380-0.972	0.038	0.62	0.336-1.152	0.131
Hemoglobin count	0.99	0.978-1.008	0.338	1.00	0.967-1.011	0.314
Leukocyte count	1.02	0.921-1.131	0.697	1.99	0.090-43.955	0.663
Monocyte count	0.93	0.250-3.471	0.916	0.12	0.003-4.048	0.236
Lymphocyte count	1.18	0.909-1.529	0.213	0.74	0.032-16.965	0.849
Neutrophile count	0.96	0.882-1.053	0.415	0.52	0.023-11.719	0.682
Basophile count	1.01	0.030-33.474	0.998	0.77	0.006-96.172	0.915
Eosinophile count	1.20	0.439-3.249	0.727	0.62	0.020-18.891	0.785

In multivariable analysis, all variables were simultaneously present in the analysis.
TPY, tobacco pack years; PAASI, PhotoAging Area and Severity Index; BMI, body mass index; UV, ultraviolet; IS, immunosuppression; Ref., reference.

Supplementary table S3. The logistic regression analysis and consequent Odds Ratios for subjects with a history of melanoma compared to control subjects without melanoma in 488 subjects at Kuopio University Hospital between May 2017 and October 2020.

Variable	Simple Odds Ratio	95% Confidence interval	P value	Multivariable Odds Ratio	95% Confidence interval	P value
Pack years:						
Never smoker	Ref., 1			Ref., 1		
≤10 TPY	0.74	0.378-1.455	0.384	0.84	0.363-1.924	0.673
>10 TPY	0.71	0.387-1.317	0.281	0.82	0.383-1.765	0.615
PAASI score	1.00	0.999-1.008	0.160	1.00	0.996-1.010	0.426
Age	1.01	0.989-1.022	0.509	1.02	0.991-1.045	0.188
Gender:						
Male	Ref., 1			Ref., 1		
Female	0.91	0.591-1.407	0.677	1.31	0.628-2.711	0.475
BMI	1.02	0.981-1.069	0.283	1.03	0.977-1.089	0.263
Mole count:						
0-20	Ref.			Ref.		
21-50	2.56	1.469-4.448	<.001	3.56	1.737-7.291	<.001
51-100	2.77	1.501-5.120	0.001	3.53	1.589-7.830	0.002
>100	2.28	1.412-5.477	0.003	3.83	1.626-9.033	0.002
Lifetime sun exposure:						
Very seldom	Ref., 1			Ref., 1		
Occasionally	0.75	0.404-1.377	0.348	0.65	0.297-1.409	0.272
Often	0.76	0.401-1.450	0.409	0.56	0.233-1.357	0.200
Very often	1.15	0.564-2.352	0.699	1.00	0.364-2.760	0.996
Main working environment:						
Outdoors	Ref., 1			Ref., 1		
Indoors	0.49	0.235-1.032	0.060	0.61	0.213-1.766	0.364
Both variably	0.52	0.227-1.171	0.113	0.69	0.232-2.069	0.511
Lifetime sunburns:						
Seldom	Ref., 1			Ref., 1		
Occasionally	1.30	0.762-2.218	0.336	1.40	0.719-2.706	0.325
Often	1.95	1.071-3.551	0.029	1.68	0.690-4.107	0.253
Solarium:						
Never	Ref., 1			Ref., 1		
0-30	1.15	0.693-1.914	0.586	1.11	0.573-2.130	0.766
31-100	1.00	0.393-2.530	0.994	0.81	0.235-2.780	0.735
UV light treatment:						
Never	Ref., 1			Ref., 1		
0-30	0.40	0.118-1.339	0.137	0.26	0.057-1.219	0.088
31-100	0.62	0.136-2.817	0.536	0.30	0.035-2.557	0.271
Skin phototype (Fitzpatrick):						
1	Ref., 1			Ref., 1		
2	0.43	0.176-1.052	0.064	0.84	0.242-2.926	0.786
3	0.37	0.152-0.903	0.029	0.75	0.203-2.750	0.661
4	0.71	0.202-2.528	0.602	1.93	0.350-10.617	0.451
Immunosuppression:						
Non-IS	Ref., 1			Ref., 1		
IS	0.39	0.196-0.786	0.008	0.37	0.148-0.912	0.031
Hemoglobin count	1.02	0.998-1.035	0.076	1.01	0.985-1.041	0.377
Leukocyte count	1.01	0.894-1.148	0.835	8.20	0.214-313.497	0.258
Monocyte count	1.15	0.233-5.649	0.865	0.35	0.005-23.468	0.625
Lymphocyte count	0.85	0.606-1.187	0.336	0.08	0.002-3.194	0.178
Neutrophile count	1.00	0.928-1.068	0.902	0.13	0.003-4.851	0.265
Basophile count	1.85	0.027-127.976	0.775	0.71	0.002-244.004	0.908
Eosinophile count	0.54	0.113-2.557	0.435	0.03	0.000-1.841	0.095

In multivariable analysis, all variables were simultaneously present in the analysis.

TPY, tobacco pack years; PAASI, PhotoAging Area and Severity Index; BMI, body mass index; UV, ultraviolet; IS, immunosuppression; Ref., reference.

Supplementary table S4. The logistic regression analysis and consequent Odds Ratios for subjects with PAASI score over median compared to control subjects with PAASI score below or equal to median in 488 subjects at Kuopio University Hospital between May 2017 and October 2020.

Variable	Simple Odds Ratio	95% Confidence interval	P value	Multivariable Odds Ratio	95% Confidence interval	P value
Pack years:						
Never smoker	Ref., 1			Ref., 1		
≤10 TPY	1.52	0.903-2.571	0.115	1.15	0.557-2.388	0.700
>10 TPY	1.54	0.960-2.479	0.073	0.92	0.484-1.746	0.798
Age	1.10	1.079-1.125	<.001	1.11	1.081-1.141	<.001
Gender:						
Male	Ref., 1			Ref., 1		
Female	0.63	0.441-9.893	0.010	1.02	0.543-1.910	0.955
BMI	0.98	0.939-1.012	0.181	0.94	0.887-0.993	0.027
Lifetime sun exposure:						
Very seldom	Ref., 1			Ref., 1		
Occasionally	1.17	0.707-1.948	0.535	1.45	0.713-2.938	0.306
Often	1.65	0.971-2.811	0.064	1.38	0.624-3.044	0.427
Very often	2.24	1.195-4.206	0.012	2.85	1.045-7.784	0.041
Main working environment:						
Outdoors	Ref., 1			Ref., 1		
Indoors	0.46	0.225-0.936	0.032	1.14	0.423-3.068	0.797
Both variably	0.80	0.371-1.734	0.576	1.24	0.439-3.477	0.690
Lifetime sunburns:						
Seldom	Ref., 1			Ref., 1		
Occasionally	0.66	0.438-0.994	0.047	0.79	0.452-1.384	0.412
Often	1.30	0.789-2.131	0.305	2.01	0.901-4.473	0.088
Solarium:						
Never	Ref., 1			Ref., 1		
0-30	1.12	0.732-1.701	0.610	1.69	0.922-3.110	0.089
31-100	0.83	0.388-1.779	0.634	0.96	0.313-2.966	0.949
UV light treatment:						
Never	Ref., 1			Ref., 1		
0-30	2.01	0.942-4.306	0.071	2.07	0.736-5.840	0.167
31-100	1.48	0.504-4.329	0.477	1.73	0.422-7.064	0.447
Skin phototype (Fitzpatrick):						
1	Ref., 1			Ref., 1		
2	0.77	0.325-1.833	0.558	1.19	0.375-3.746	0.772
3	0.93	0.395-2.200	0.874	1.56	0.465-5.222	0.473
4	1.26	0.371-4.287	0.711	1.53	0.290-8.105	0.616
Immunosuppression:						
Non-IS	Ref., 1			Ref., 1		
IS	0.70	0.446-1.104	0.125	0.81	0.427-1.552	0.533
Hemoglobin count	1.01	0.994-1.024	0.229	1.01	0.982-1.032	0.605
Leukocyte count	1.05	0.949-1.165	0.337	0.88	0.469-1.659	0.696
Monocyte count	9.85	2.477-39-196	0.001	7.64	0.701-83.164	0.095
Lymphocyte count	0.96	0.744-1.243	0.765	1.01	0.469-2.175	0.979
Neutrophile count	1.05	0.954-1.154	0.322	1.12	0.607-2.050	0.726
Basophile count	7.40	0.230-237.272	0.258	15.86	0.105-2389.402	0.280
Eosinophile count	1.08	0.397-2.909	0.887	0.46	0.061-3.446	0.448

In multivariable analysis, all variables were simultaneously present in the analysis.
TPY, tobacco pack years; PAASI, PhotoAging Area and Severity Index; BMI, body mass index; UV, ultraviolet; IS, immunosuppression; Ref., reference.

Supplementary table S5. The logistic regression analysis and consequent Odds Ratios for subjects with facial photoaging score 3-4 compared to control subjects with score 0-2 in 488 subjects at Kuopio University Hospital between May 2017 and October 2020.

Variable	Simple Odds Ratio	95% Confidence interval	P value	Multivariable Odds Ratio	95% Confidence interval	P value
Pack years:						
Never smoker	Ref., 1			Ref., 1		
≤10 TPY	1.80	1.042-3.109	0.035	1.22	0.571-2.596	0.611
>10 TPY	1.75	1.067-2.882	0.027	1.09	0.551-2.156	0.806
Age	1.15	1.109-1.183	<.001	1.14	1.098-1.184	<.001
Gender:						
Male	Ref., 1			Ref., 1		
Female	0.59	0.399-0.870	0.008	1.22	0.619-2.404	0.566
BMI	1.03	0.987-1.068	0.188	1.03	0.973-1.082	0.348
Lifetime sun exposure:						
Very seldom	Ref., 1			Ref., 1		
Occasionally	0.74	0.430-1.255	0.259	0.70	0.335-1.470	0.347
Often	0.70	0.400-1.234	0.219	0.59	0.260-1.350	0.213
Very often	0.78	0.404-1.507	0.460	0.76	0.271-2.136	0.604
Main working environment:						
Outdoors	Ref., 1			Ref., 1		
Indoors	0.56	0.274-1.129	0.104	1.40	0.502-3.901	0.520
Both variably	1.01	0.472-2.152	0.984	1.55	0.540-4.473	0.414
Lifetime sunburns:						
Seldom	Ref., 1			Ref., 1		
Occasionally	0.73	0.467-1.128	0.154	0.92	0.499-1.681	0.778
Often	0.81	0.479-1.380	0.443	1.35	0.583-3.135	0.483
Solarium:						
Never	Ref., 1			Ref., 1		
0-30	0.54	0.328-0.895	0.017	0.67	0.342-1.299	0.233
31-100	0.63	0.264-1.517	0.305	0.85	0.249-2.876	0.788
UV light treatment:						
Never	Ref., 1			Ref., 1		
0-30	1.56	0.736-3.312	0.246	1.36	0.512-3.587	0.541
31-100	0.67	0.185-2.457	0.550	0.32	0.053-1.925	0.214
Skin phototype (Fitzpatrick):						
1	Ref., 1			Ref., 1		
2	0.71	0.296-1.722	0.454	0.78	0.232-2.641	0.693
3	0.61	0.255-1.468	0.271	0.75	0.209-2.667	0.653
4	0.90	0.261-3.088	0.864	0.73	0.126-4.213	0.724
Immunosuppression:						
Non-IS	Ref., 1			Ref., 1		
IS	0.77	0.463-1.279	0.313	0.73	0.351-1.521	0.401
Hemoglobin count	1.00	0.987-1.019	0.688	1.01	0.985-1.037	0.412
Leukocyte count	1.12	1.004-1.251	0.043	1.14	0.683-1.901	0.617
Monocyte count	13.21	3.245-53.788	<.001	2.80	0.254-30.762	0.401
Lymphocyte count	1.14	0.864-1.496	0.359	0.75	0.378-1.481	0.404
Neutrophile count	1.00	0.949-1.062	0.892	0.94	0.585-1.502	0.787
Basophile count	4.75	0.110-205.158	0.417	2.92	0.015-582.871	0.691
Eosinophile count	1.24	0.436-3.503	0.691	0.24	0.031-1.817	0.166

In multivariable analysis, all variables were simultaneously present in the analysis.
TPY, tobacco pack years; PAASI, PhotoAging Area and Severity Index; BMI, body mass index; UV, ultraviolet; IS, immunosuppression; Ref., reference.

Supplementary table S6. The logistic regression analysis and consequent Odds Ratios for subjects with one or more actinic keratoses compared to control subjects without actinic keratosis in 488 subjects at Kuopio University Hospital between May 2017 and October 2020.

Variable	Simple Odds Ratio	95% Confidence interval	P value	Multivariable Odds Ratio	95% Confidence interval	P value
Pack years:						
Never smoker	Ref., 1			Ref., 1		
≤10 TPY	2.17	1.279-3.679	0.004	1.39	0.624-3.074	0.423
>10 TPY	1.21	0.754-1.939	0.430	0.56	0.279-1.124	0.103
Age	1.15	1.118-1.182	<.001	1.16	1.119-1.202	<.001
Gender:						
Male	Ref., 1			Ref., 1		
Female	0.43	0.301-0.619	<.001	0.53	0.268-1.034	0.062
BMI	1.05	1.006-1.086	0.023	1.04	0.988-1.098	0.128
Lifetime sun exposure:						
Very seldom	Ref., 1			Ref., 1		
Occasionally	0.75	0.455-1.250	0.274	0.72	0.333-1.562	0.406
Often	1.07	0.634-1.810	0.798	0.82	0.345-1.941	0.648
Very often	0.85	0.459-1.577	0.607	0.93	0.314-2.728	0.889
Main working environment:						
Outdoors	Ref., 1			Ref., 1		
Indoors	0.25	0.119-0.521	<.001	0.43	0.147-1.275	0.128
Both variably	0.54	0.246-1.197	0.130	0.64	0.206-1.965	0.432
Lifetime sunburns:						
Seldom	Ref., 1			Ref., 1		
Occasionally	0.65	0.429-0.973	0.037	0.91	0.494-1.668	0.756
Often	0.82	0.504-1.343	0.435	1.58	0.671-3.701	0.297
Solarium:						
Never	Ref., 1			Ref., 1		
0-30	0.94	0.616-1.435	0.774	1.88	0.976-3.631	0.059
31-100	0.83	0.387-1.768	0.624	2.25	0.709-7.137	0.169
UV light treatment:						
Never	Ref., 1			Ref., 1		
0-30	2.40	1.123-5.134	0.024	2.42	0.830-7.078	0.106
31-100	0.53	0.163-1.711	0.287	0.32	0.064-1.587	0.162
Skin phototype (Fitzpatrick):						
1	Ref., 1			Ref., 1		
2	0.72	0.310-1.691	0.456	1.07	0.302-3.750	0.922
3	0.79	0.341-1.834	0.584	1.60	0.425-6.005	0.487
4	0.82	0.249-2.690	0.741	0.96	0.155-5.866	0.960
Immunosuppression:						
Non-IS	Ref., 1			Ref., 1		
IS	0.54	0.337-0.863	0.010	0.58	0.285-1.187	0.136
Hemoglobin count	1.01	0.997-1.027	0.126	1.01	0.982-1.035	0.537
Leukocyte count	1.12	1.010-1.243	0.031	1.18	0.883-1.569	0.266
Monocyte count	11.07	2.843-43.106	<.001	1.19	0.097-14.625	0.891
Lymphocyte count	1.09	0.840-1.406	0.526	1.20	0.697-2.072	0.509
Neutrophile count	1.00	0.947-1.055	0.987	0.95	0.775-1.173	0.654
Basophile count	1.21	0.037-38.900	0.916	0.11	0.000-22.634	0.411
Eosinophile count	1.25	0.460-3.409	0.659	0.14	0.017-1.180	0.071

In multivariable analysis, all variables were simultaneously present in the analysis.

TPY, tobacco pack years; PAASI, PhotoAging Area and Severity Index; BMI, body mass index; UV, ultraviolet; IS, immunosuppression; Ref., reference.

Supplementary table S7. The logistic regression analysis and consequent Odds Ratios for subjects with over 50 moles compared to control subjects with 0-50 moles in 488 subjects at Kuopio University Hospital between May 2017 and October 2020.

Variable	Simple Odds Ratio	95% Confidence interval	P value	Multivariable Odds Ratio	95% Confidence interval	P value
Pack years:						
Never smoker	Ref., 1			Ref., 1		
≤10 TPY	0.79	0.441-1.426	0.439	1.32	0.637-2.739	0.455
>10 TPY	0.49	0.273-0.888	0.019	0.86	0.419-1.763	0.680
PAASI score	0.99	0.984-0.994	<.001	1.01	0.998-1.011	0.194
Age	0.94	0.925-0.955	<0.001	0.93	0.903-0.947	<.001
Gender:						
Male	Ref., 1			Ref., 1		
Female	0.94	0.639-1-395	0.774	0.75	0.375-1.482	0.402
BMI	1.02	0.982-1.064	0.285	1.06	1.011-1.120	0.017
Lifetime sun exposure:						
Very seldom	Ref., 1			Ref., 1		
Occasionally	0.79	0.453-1.371	0.400	0.45	0.216-0.953	0.037
Often	0.81	0.452-1.440	0.467	0.53	0.232-1.188	0.122
Very often	0.95	0.488-1.857	0.885	0.72	0.266-1.950	0.519
Main working environment:						
Outdoors	Ref., 1			Ref., 1		
Indoors	1.54	0.680-3.481	0.301	0.53	0.189-1.499	0.233
Both variably	1.10	0.453-2.686	0.829	0.57	0.190-1.692	0.309
Lifetime sunburns:						
Seldom	Ref., 1			Ref., 1		
Occasionally	1.92	1.188-3.097	0.008	1.85	0.993-3.461	0.053
Often	1.48	0.830-2.625	0.185	1.12	0.481-2.624	0.788
Solarium:						
Never	Ref., 1			Ref., 1		
0-30	1.53	0.968-2.404	0.069	1.76	0.946-3.289	0.074
31-100	1.26	0.556-2.845	0.583	0.85	0.274-2.618	0.774
UV light treatment:						
Never	Ref., 1			Ref., 1		
0-30	0.48	0.179-1.277	0.141	0.53	0.158-1.772	0.302
31-100	0.18	0.024-1.420	0.104	0.17	0.018-1.548	0.115
Skin phototype (Fitzpatrick):						
1	Ref., 1			Ref., 1		
2	1.07	0.420-2.702	0.893	0.82	0.246-2.750	0.751
3	0.84	0.331-2.121	0.709	0.87	0.251-3.013	0.826
4	0.81	0.212-3.096	0.757	1.20	0.215-6.710	0.835
Immunosuppression:						
Non-IS	Ref., 1			Ref., 1		
IS	0.43	0.236-0.770	0.005	0.30	0.138-0.651	0.002
Hemoglobin count	1.01	0.997-1.030	0.102	1.01	0.983-1.036	0.493
Leukocyte count	0.89	0.785-0.997	0.044	0.88	0.467-1.671	0.703
Monocyte count	0.19	0.041-0.924	0.040	0.68	0.054-8.498	0.765
Lymphocyte count	0.81	0.593-1.095	0.167	0.78	0.354-1.733	0.546
Neutrophile count	1.02	0.967-1.080	0.443	1.13	0.614-2.067	0.701
Basophile count	0.17	0.003-8.201	0.366	0.03	0.000-7.623	0.214
Eosinophile count	0.68	0.193-2.418	0.554	1.56	0.395-6.174	0.525

In multivariable analysis, all variables were simultaneously present in the analysis.
TPY, tobacco pack years; PAASI, PhotoAging Area and Severity Index; BMI, body mass index; UV, ultraviolet; IS, immunosuppression; Ref., reference.

© 2024 Uotila I. et al.
